# SOX9 promotes tumor progression through the axis BMI1-p21^CIP^

**DOI:** 10.1038/s41598-019-57047-w

**Published:** 2020-01-15

**Authors:** Paula Aldaz, Maddalen Otaegi-Ugartemendia, Ander Saenz-Antoñanzas, Mikel Garcia-Puga, Manuel Moreno-Valladares, Juana M. Flores, Daniela Gerovska, Marcos J. Arauzo-Bravo, Nicolas Samprón, Ander Matheu, Estefania Carrasco-Garcia

**Affiliations:** 1 Cellular Oncology Group, Biodonostia Health Research Institute, San Sebastian, Spain; 2 Donostia Hospital, San Sebastian, Spain; 3 Department of Animal Medicine and Surgery, Complutense University of Madrid, Madrid, Spain; 4 Computational Biology and Systems Biomedicine Group, Biodonostia Health Research Institute, San Sebastian, Spain; 5 CIBERfes, Madrid, Spain; 6 IKERBASQUE, Basque Foundation for Science, Bilbao, Spain

**Keywords:** Mechanisms of disease, Tumour heterogeneity

## Abstract

The developmental regulator SOX9 is linked to cancer progression mainly as a result of its role in the regulation of cancer stem cells (CSCs). However, its activity in the differentiated cells that constitute the heterogeneous tumor bulk has not been extensively studied. In this work, we addressed this aspect in gastric cancer, glioblastoma and pancreatic adenocarcinoma. *SOX9* silencing studies revealed that SOX9 is required for cancer cell survival, proliferation and evasion of senescence *in vitro* and tumor growth *in vivo*. Gain of-*SOX9* function showed that high levels of SOX9 promote tumor cell proliferation *in vitro* and *in vivo*. Mechanistically, the modulation of SOX9 changed the expression of the transcriptional repressor BMI1 in the same direction in the three types of cancer, and the expression of the tumor suppressor p21^CIP^ in the opposite direction. In agreement with this, SOX9 expression positively correlated with BMI1 levels and inversely with p21^CIP^ in clinical samples of the different cancers. Moreover, BMI1 re-establishment in *SOX9*-silenced tumor cells restored cell viability and proliferation as well as decreased p21^CIP^ *in vitro* and tumor growth *in vivo*. These results indicate that BMI1 is a critical effector of the pro-tumoral activity of SOX9 in tumor bulk cells through the repression of p21^CIP^. Our results highlight the relevance of the SOX9-BMI1-p21^CIP^ axis in tumor progression, shedding novel opportunities for therapeutic development.

## Introduction

Some types of cancer exhibit a dismal prognosis, mainly due to its late diagnosis, its aggressive progression and the resistance to the available chemotherapy, in which inter and intra-tumoral heterogeneity play a major role^1,2^. One of these types of cancer is pancreatic ductal adenocarcinoma (PDAC), with a 5-year survival rate of less than 6%^3^ and very similar rates of incidence and mortality (458,918 new diagnoses and 432,242 deaths worldwide in 2018)^4^. Glioblastoma (GBM), the most malignant primary brain tumor, also represents a challenge in oncology despite having a low incidence (less than 5 cases per 100,000 people), as exhibits a 5-year survival rate of 5.6%^5^ and a median survival of 14.6 months^6^. For its part, gastric cancer (GC) is the third leading cause of cancer deaths worldwide, with an estimated 783,000 deaths in 2018^4^.

*SOX9* [SRY (Sex determining region Y)-box 9] is a member of the SOX family of transcription factors, which are developmental regulators characterized by a conserved high mobility group (HMG) DNA-binding domain^7^. SOX9 regulates stem cell maintenance and instruction of cell fate, and exerts relevant roles during development, such as sex determination, neural crest development, chondrogenesis or pancreas development^8–10^. During embryogenesis, SOX9 is expressed and regulates progenitor proliferation and differentiation, being required for maintaining tissue identity in different contexts, but mainly in the brain and gastrointestinal system^10–12^. Likewise, in the adulthood, SOX9 also plays a relevant role in the maintenance of the homeostasis of these tissues through the regulation of the residing populations of adult stem cells^11,13^, although not exclusively, as its expression has also been linked to several differentiated cells within different contexts and tissues^8,14^.

In cancer, several studies demonstrated the involvement of SOX9 in cancer formation, as the elevation of its levels favors transformation of stem cells. For example in pancreas, where SOX9 regulates pancreatic progenitor cells during pancreas development and maintains ductal integrity in mature pancreas^15,16^, it is essential during acinar to ductal metaplasia (ADM) initiation^17^ and has been shown indispensable for the formation of intraepithelial neoplasias (PanINs) induced by oncogenic *KRAS*^18^. Similar observations have been observed in the brain, breast or skin^19–22^. Moreover, several studies have linked SOX9 to cancer initiation and the regulation of the population of cancer stem cells (CSCs) in multiples tissues^19,21,23^, promoting processes associated to this specific population such as metastasis or chemoresistance^24–27^.

In clinical samples, SOX9 expression is elevated in glioblastoma, pancreatic ductal adenocarcinoma, gastric cancer, colon, skin or breast cancer samples respect to adjacent normal tissue^28–30^. Furthermore, high levels of SOX9 have been associated with tumor grade, dismal prognosis and poor patient survival in patients of those types of cancer^28,31,32^. Thus, SOX9 expression and function are altered in diverse human cancers, acting as an oncogene in a wide range of them, mainly through the regulation of CSCs activity, and as a tumor suppressor in specific situations^26^. Besides, it has been shown that Sox9 is able to promote proliferation and induce neoplastic transformation of primary fibroblasts^33^, indicative that SOX9 is relevant in cancer beyond its initiation and its role in CSC activity. In this regard, its activity in cells of tumor bulk and the underlying molecular mechanisms remain poorly understood. Therefore, in this study we elucidated the functional role of SOX9 in critical processes for cancer progression such as survival, proliferation and senescence in tumor differentiated cells, and deciphered its molecular mechanism, providing new knowledge regarding the role and molecular pathway of this critical stem cell factor on cancer progression and heterogeneity.

## Results

### SOX9 is required for tumor cell survival and proliferation

**Figure 1:**
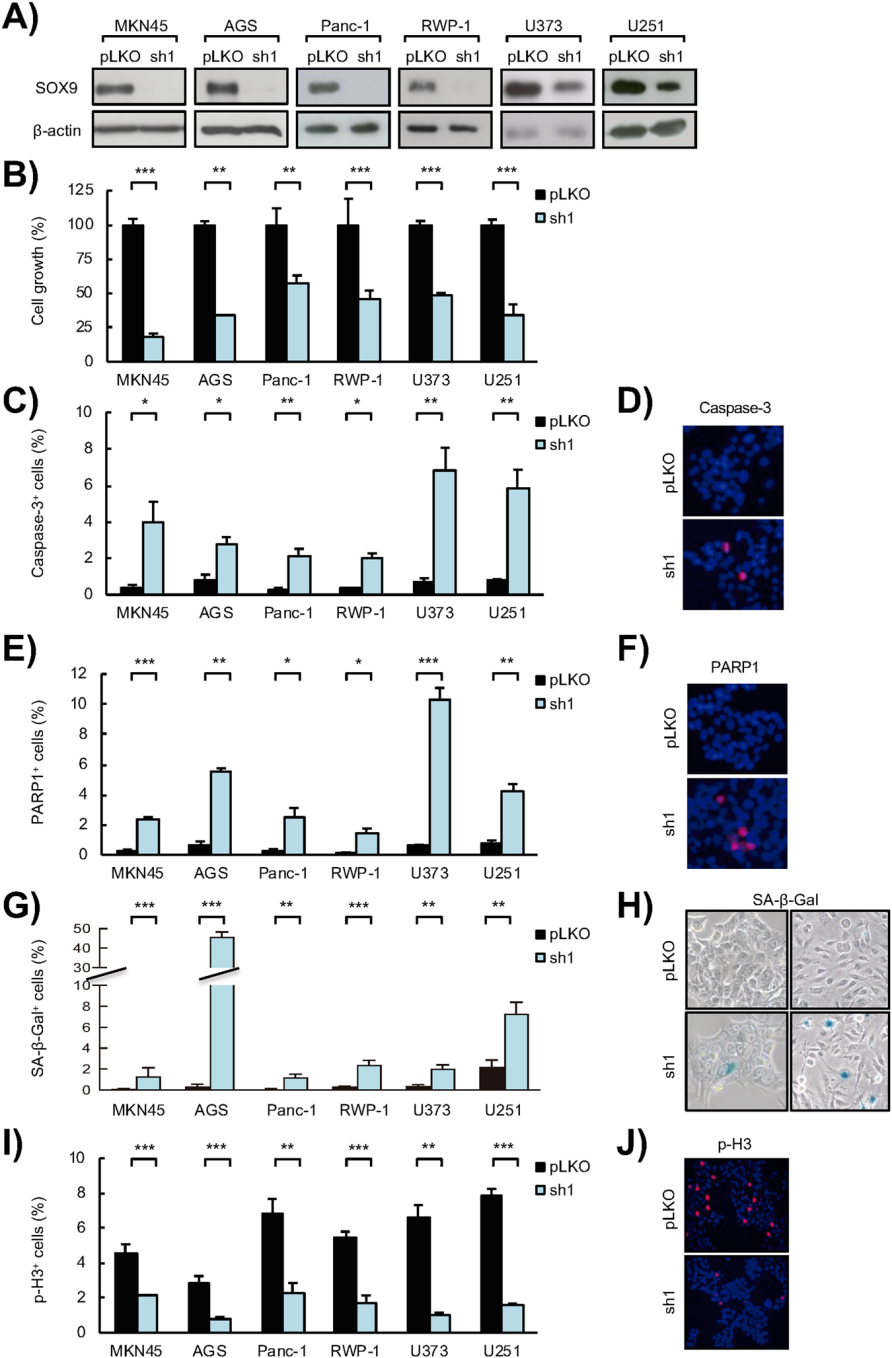
*SOX9* silencing impairs tumor cell survival, induces senescence and abrogates proliferation in cancer cells. **(A)** Representative Western blots of SOX9 protein expression in MKN45 and AGS GC cell lines, Panc-1 and RWP-1 PDAC cell lines, and U373 and U251 GBM cell lines lentivirally transduced with a specific shRNA against *SOX9* (*sh1*) or the corresponding control vector (*pLKO*) (n ≥ 4). β-actin levels were used as a loading control. (**B)** Cell growth at day 5 (percentage of cells) in *SOX9*-silenced (*sh1*) cells respect to control (*pLKO*) cells (n ≥ 4). (**C)** Apoptosis represented by the percentage of active Caspase-3 positive cells in *SOX9*-silenced (*sh1*) cells and the corresponding control cells (*pLKO*) determined by immunofluorescence staining (n ≥ 4). (**D)** Representative image of active Caspase-3 positive cells in RWP-1 control (*pLKO*) and SOX9-silenced (*sh1*) cells. (**E)** Apoptosis represented by the percentage of cleaved PARP1 positive cells in *SOX9*-silenced (*sh1*) cells and the corresponding control cells (*pLKO*) determined by immunofluorescence staining (n ≥ 4). (**F)** Representative image of cleaved PARP1 in RWP-1 control (*pLKO*) and *SOX9*-silenced (*sh1*) cells. **(G)** Senescence represented as the quantification of the percentage of β-Galactosidase (SA β-Gal) positive cells in *SOX9*-silenced (*sh1*) cells and the corresponding control cells (*pLKO*) (n ≥ 4). (**H)** Representative images of SA β-Gal staining in RWP-1 (left) and U251 (right) control (*pLKO*) and *SOX9*-silenced (*sh1*) cells. (**I)** Proliferative capacity represented by the percentage of phospho-histone H3 (p-H3) positive cells (n ≥ 4) determined by immunofluorescence staining in control (*pLKO*) and *SOX9*-silenced (*sh1*) cells. (**J)** Representative image of p-H3 in RWP-1 control (*pLKO*) and *SOX9*-silenced (*sh1*) cells. Asterisks (*, ** and ***) indicate statistical significance (p < 0.05, p < 0.01, and p < 0.001, respectively).

In order to assess the impact of SOX9 in tumor cell survival, we knocked down *SOX9* expression in different cancer cell lines. In particular, we silenced *SOX9* expression in cell lines of gastric cancer (AGS and MKN45), pancreatic cancer (Panc-1 and RWP-1) and glioblastoma (U373 and U251), which exhibit high SOX9 expression levels. After confirming the successful reduction of SOX9 levels in these cell lines (Fig. 1A and Fig. Suppl), we determined cell viability by cell count experiments. In these analyses, we observed a significantly reduced number of cells in *SOX9*-silenced cultures respect to control cells 5 days after the seeding (Fig. 1B), indicating that *SOX9* silencing compromises the viability of tumor cells.

In order to evaluate the role of SOX9 in cancer cell survival, we studied apoptosis. For this, we analyzed the activation of Caspase-3 and the proteolytic inactivation of PARP1 through immunofluorescence staining. These experiments revealed the presence of a significantly higher number of apoptotic cells in cultures with *SOX9* silencing, with a marked increase of over 10 fold in both active Caspase-3 (Fig. 1C,D) and cleaved PARP1-positive cells (Fig. 1E,F) in *SOX9*-silenced cells respect to controls. Thus, SOX9 is required for cancer cell survival, wherein exerts an antiapoptotic role.

Given that cellular senescence constitutes a tumor-suppressive mechanism that restricts tumor progression, we explored whether SOX9 would mediate senescence evasion in cancer cells. To assess this aspect, we analyzed the senescence associated β-galactosidase activity in *SOX9*-silenced cells. We detected a significant increase in the number of β-galactosidase-positive cells in the different cancer types (Fig. 1G,H), revealing that *SOX9* silencing promotes the induction of senescence in cancer cells.

**Figure 2:**
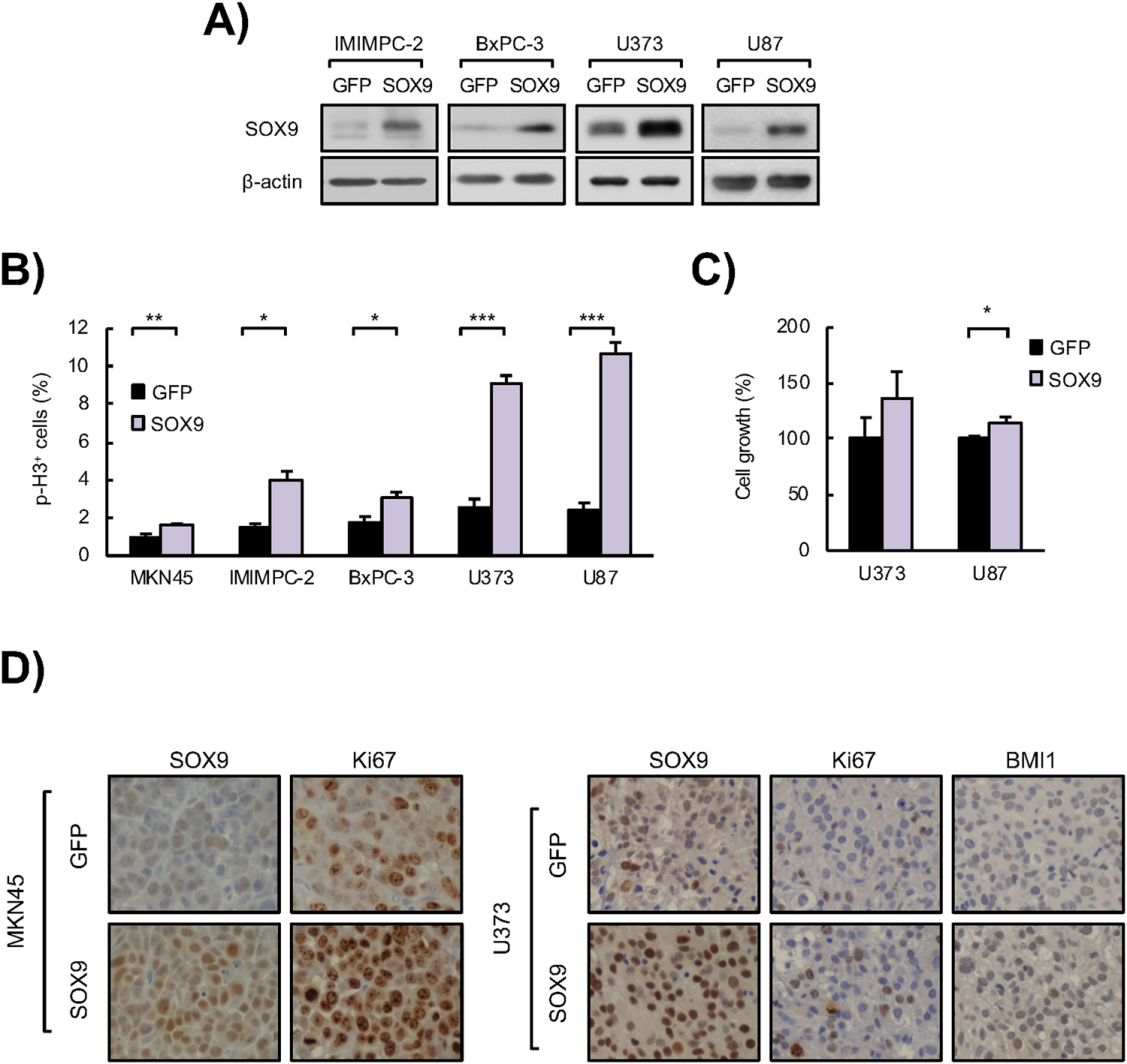
SOX9 ectopic upregulation enhances tumor cell proliferation. (**A**) Representative Western blots of SOX9 protein expression in IMIMPC-2 and BxPC-3 PDAC cell lines, and U373 and U87 GBM cell lines lentivirally transduced with plasmids harboring *GFP* (*GFP*) or *SOX9* (*SOX9*) coding sequences (n ≥ 3). (**B)** Quantification of the number of p-H3 positive cells in *SOX9* transduced cells compared to control cells (*GFP*) (n ≥ 4). (**C)** Cell growth at day 5 (percentage of cells) comparing control (*GFP*) and *SOX9* overexpressing U373 and U87 GBM cells (n ≥ 3). (**D)** Representative images of SOX9, BMI1 and Ki67 protein expression determined by immunohistochemistry in subcutaneous tumors generated in nude mice by injection of *SOX9* overexpressing (*SOX9*) and control (*GFP*) MKN45 (left) and U373 (right) cells. Asterisks (*, ** and ***) indicate statistical significance (p < 0.05, p < 0.01, and p < 0.001, respectively).

Next, we measured cell proliferation through the evaluation of the percentage of cells positive for the marker of mitosis phospho-Histone H3 (p-H3). Our results revealed a marked and significant decrease in mitotic cells in *SOX9*-silenced cancer cells respect to cells transduced with empty vector (Fig. 1I,J). On the contrary, ectopic upregulation of *SOX9* in cancer cell lines (Fig. 2A) resulted in a significant increase in the percentage of p-H3 positive cells in cultures from the 3 types of cancer (Fig. 2B), as well as increased cell count (Fig. 2C). In line with this, tumors derived from MKN45 gastric cancer cells and U373 glioma cells with overexpression of SOX9 presented a markedly higher number of Ki67 positive cells than those tumors formed by control cells *in vivo* (Fig. 2D), together demonstrating that SOX9 regulates cancer cell proliferation.

### SOX9 expression regulates BMI1 and p21^CIP^ levels

Next, we wanted to unravel the molecular mechanism underlying the role of SOX9 in cell survival and proliferation. Since we had previously found that *Sox9* promotes proliferation and facilitates neoplastic transformation of primary fibroblasts via the transcriptional repressor *Bmi1*^33^, and other groups have shown that SOX9 induces cancer cell proliferation through downregulation of the tumor suppressor p21^CIP ^^34,35^, we studied their expression.

**Figure 3:**
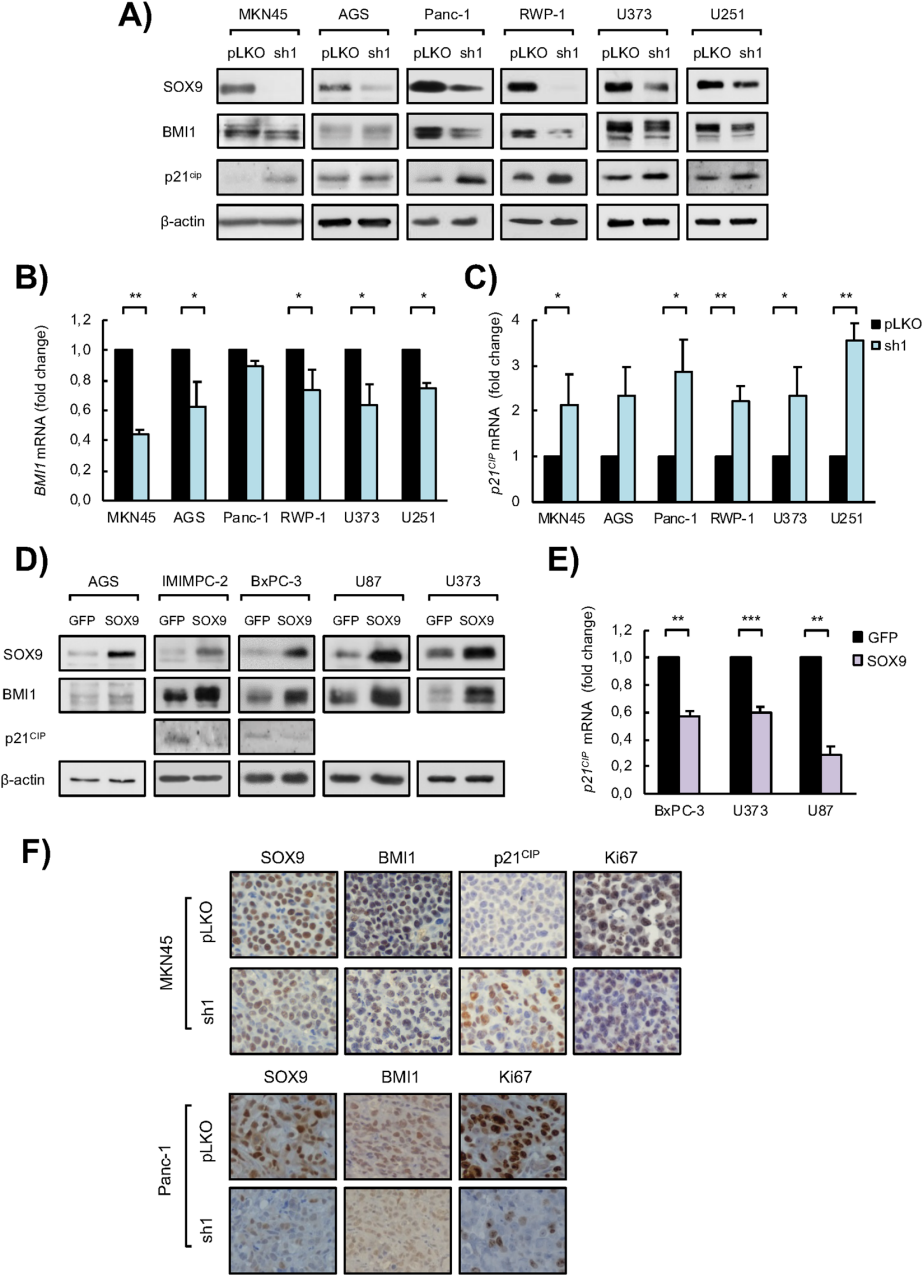
*SOX9* modulation impacts on *BMI1* and *p21*^*CIP*^ expression in cancer cells. (**A)** Representative Western blots of SOX9, BMI1 and p21^CIP^ protein expression in MKN45 and AGS GC cell lines, Panc-1 and RWP-1 PDAC cell lines, and U373 and U251 GBM cell lines lentivirally transduced with a specific shRNA against *SOX9* (*sh1*) or the corresponding control vector (*pLKO*) (n ≥ 3). β-actin levels were used as a loading control. (**B)** *BMI1* mRNA levels in control (*pLKO*) and SOX9-silenced (*sh1*) cells in the indicated GC, PDAC and GBM cell lines (n ≥ 2). (**B)** *p21*^*CIP*^ mRNA levels in control (*pLKO*) and SOX9-silenced (*sh1*) cells in the indicated GC, PDAC and GBM cell lines (n ≥ 2). (**D)** Representative Western blots of the indicated proteins in AGS GC cell line, IMIMPC-2 and BxPC-3 PDAC cell lines, and U87 and U373 GBM cell lines lentivirally transduced with plasmids harboring *GFP* (*GFP*) or *SOX9* (*SOX9*) coding sequences (n ≥ 3). β-actin levels were used as a loading control. (**E**) *p21*^*CIP*^ mRNA levels in *SOX9* overexpressing (*SOX9*) or control (*GFP*) cells in the indicated PDAC and GBM cell lines (n ≥ 2). (**F)** Representative images of SOX9, BMI1, p21^CIP^ and Ki67 protein expression determined by immunohistochemistry in subcutaneous tumors generated in nude mice by injection of MKN45 (upper panel) and Panc-1 (bottom panel) control (*pLKO*) and *SOX9*-silenced (*sh1*) cells. Asterisks (*, ** and ***) indicate statistical significance (p < 0.05, p < 0.01, and p < 0.001, respectively).

We first investigated the effect of *SOX9* silencing in their expression in the different tumor cell lines of various origins. Our results revealed that BMI1 protein expression was reduced in *SOX9*-silenced cells of PDAC, GBM and GC (Fig. 3A), whereas p21^CIP^ levels were elevated (Fig. 3A). Similar effect was also observed at transcriptional level (Fig. 3B,C). On the contrary, cells with ectopic *SOX9* overexpression displayed elevated levels of BMI1 and lower p21^CIP^ expression (Fig. 3D,E). These results show that SOX9 regulates the expression of *BMI1* and *p21*^*CIP*^ at transcriptional level in cancer cells *in vitro* and this might influence tumor cell survival and proliferation.

To further characterize the link between SOX9 expression with BMI1 and p21^CIP^, we stained tumors derived from *SOX9* silenced cells and controls with antibodies for BMI1 and p21^CIP^, as well as for SOX9 and Ki67. Thus, confirming previous results revealing that SOX9 inhibition reduces tumor growth^25,31^, immunohistochemistry analysis showed lower staining of SOX9 as well as reduction in Ki67 positive cells in gastric and pancreatic tumors with reduced SOX9 (Fig. 3F). In these contexts, BMI1 staining was lower, whereas p21^CIP^ was increased in tumors derived from *SOX9* knockdown cells (Fig. 3F). These results show that SOX9 modulates the expression of BMI1 and p21^CIP^ in different cancer types *in vivo*.

### Correlation between SOX9, BMI1 and p21^CIP^ in clinical samples

**Figure 4:**
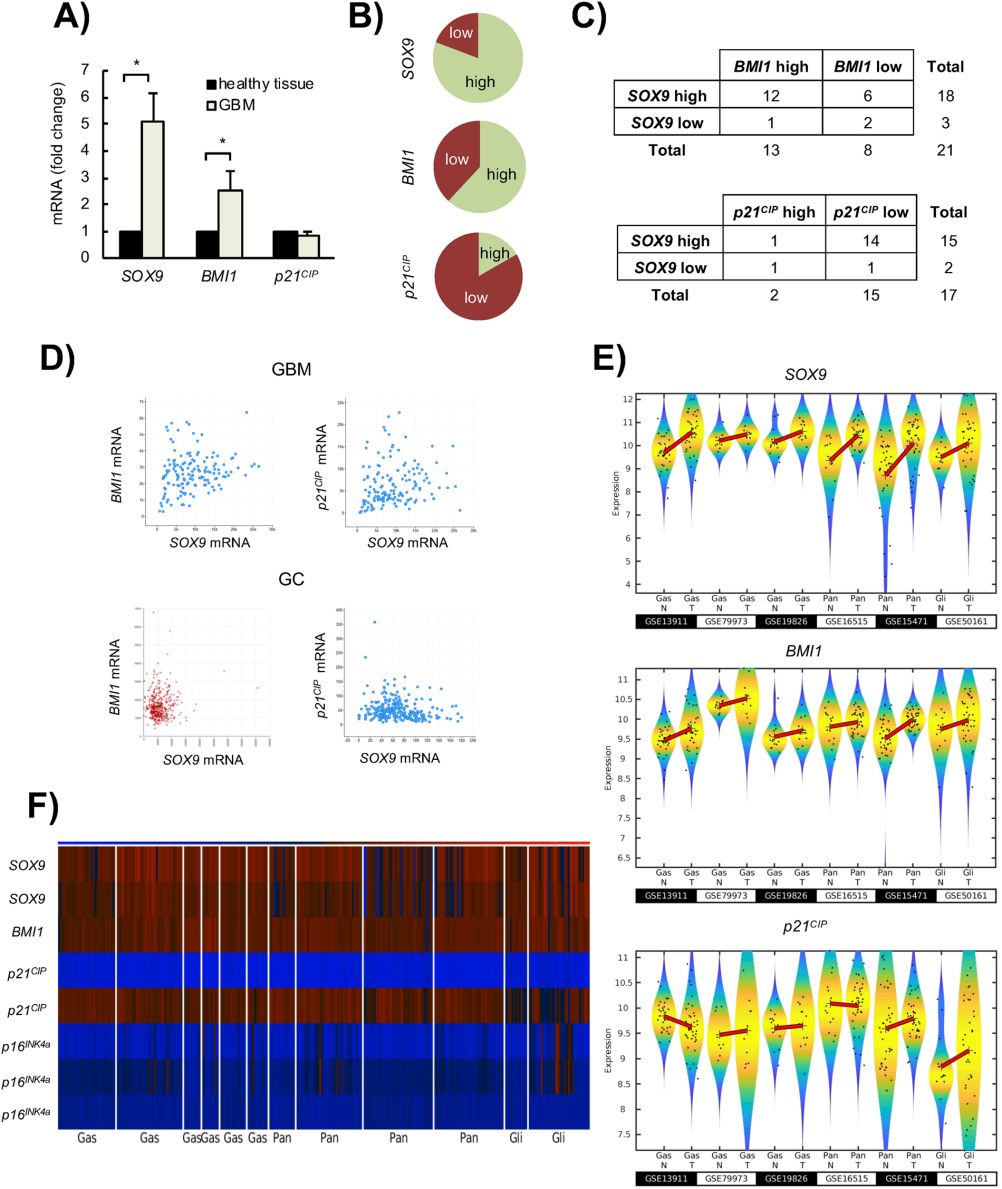
*SOX9* expression correlates positively with *BMI1* expression and negatively with *p21*^*CIP*^ expression in cancer patient samples. (**A)** *SOX9*, *BMI1* and *p21*^*CIP*^ mRNA expression levels in glioblastoma samples (GBM) from Hospital Donostia patients relative to the expression in normal brain samples (healthy tissue). (**B)** Frecuency of SOX9, *BMI1* and *p21*^*CIP*^ high and low mRNA expressing GBM samples from Hospital Donostia patients. (**C)** Association of high *SOX9* mRNA expression with high *BMI1* and low *p21*^*CIP*^ expression in human GBM samples from Hospital Donostia patients. **(D)** Graphs representing the correlation between *SOX9* and *BMI1* mRNA expression, and *SOX9* and *p21*^*CIP*^ *(CDKN1A)* mRNA expression in gastric adenocarcinoma (GC) and GBM patients from The Cancer Genome Atlas (TCGA) cohort. **GBM** (upper panel): positive correlation between *SOX9* and *BMI1* mRNA expression (Spearman’s correlation = 0.22, p-value = 8.31e-3, n = 147); and positive correlation between *SOX9* and *p21*^*CIP*^ mRNA expression (Spearman’s correlation = 0.27, p-value = 1.119e-3, n = 147). Data obtained from https://www.cancer.gov/tcga. **GC** (bottom panel): positive correlation between *SOX9* and *BMI1* mRNA expression (r-value = 0.086, p-value = 0.080, n = 450). Data obtained from *R2*: Genomics Analysis and Visualization Platform (http://r2.amc.nl); negative correlation between *SOX9* and *p21*^*CIP*^ mRNA expression (Spearman’s correlation = −0.123, p-value = 0.0478, n = 258). Data obtained from https://www.cancer.gov/tcga. (**E)** Violin plots representing the expression of *SOX9, BMI1 and p21*^*CIP*^ analyzed by microarrays in normal (N) and tumor (T) samples of three gastric cancer cohorts (datasets GSE13911, GSE79973 and GSE19826), two pancreatic adenocarcinoma cohorts (datasets GSE16515 and GSE15471) and one GBM cohort (dataset GSE50161). The black dots represent the individual expression values and the symbol + denote the mean values of the distributions. (**F)** Heatmap representing the expression of *SOX9, BMI1, p21*^*CIP*^ *(CDKN1A)* and *p16*^*Ink4a*^ *(CDKN2A)* in normal and tumor tissue samples belonging to the datasets represented in (**E**).

Next, we wondered whether the association between SOX9, BMI1 and p21^CIP^ levels could be translated to clinical samples. Therefore, we checked the relationship between their expression using previously described cohorts^25^ and available datasets from all genome-wide expression profiling arrays of glioblastoma, gastric and pancreatic cancer cohorts of patients. First, we found that *SOX9* and *BMI1* were increased by more than 5 and 2.5-fold respectively, while *p21*^*CIP*^ was not significantly altered in GBM compared to normal brain tissue (Fig. 4A). In line with this, 80% and 60% of the GBMs showed high levels of *SOX9* and *BMI1* respectively (fold change higher than 2 respect to normal brain), whereas 83% showed low or moderate expression of *p21*^*CIP*^ (Fig. 4B). Moreover, majority of the biopsies with high *SOX9* expression also presented increased levels of *BMI1* and moderate or low *p21*^*CIP*^ levels (Fig. 4C).

Next, we checked the available information obtained by The Cancer Genome Atlas (TCGA) project and found a significant correlation between *SOX9* and *BMI1* in GBM (Fig. 4D), detecting also that the expression of *SOX9* correlated positively with *BMI1* expression and negatively with *p21*^*CIP*^expression in gastric cancer (Fig. 4D). Finally, we analyzed the expression of these markers in 3 cohorts of gastric tumor-normal samples (n = 60), 2 of pancreatic tumor-normal samples (n = 124), and additional one of GBM and normal brain samples (n = 47). The comparison between normal and tumor tissue revealed frequent overexpression of *SOX9* and *BMI1*, whereas *p21*^*CIP*^ levels were similar or decreased in tumor samples (Fig. 4E). Noteworthy, the heatmap representation revealed that the expression of *SOX9* correlated positively with *BMI1* expression and negatively with *p21*^*CIP*^ in samples from the different types of cancer (Fig. 4F). Similarly, there was an inverse correlation between *SOX9* and *p16*^*INK4a*^ levels in the same samples (Fig. 4F). Importantly, these results translate the association of *SOX9-BMI1-p21*^*CIP*^ from tumor cells *in vitro* and *in vivo* tumors to clinical biopsies.

### BMI1 restoration in *SOX9*-silenced cells rescues the malignant phenotype of tumor cells

**Figure 5:**
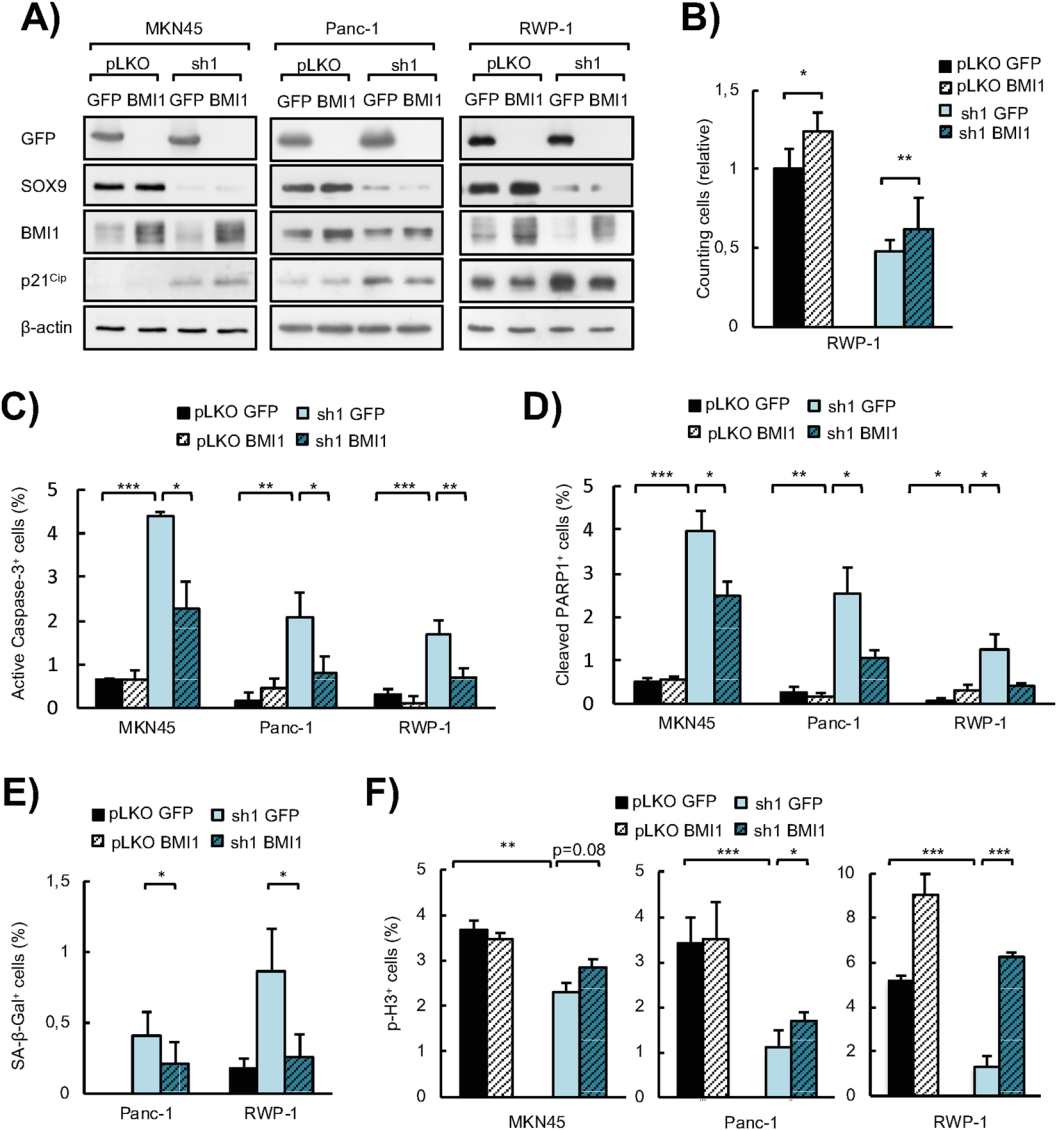
*BMI1* re-expression in cancer cells with *SOX9* silencing restores the aggressive phenotype conferred by SOX9 *in vitro*. **(A)** Representative Western blots of SOX9, BMI1, p21^CIP^ and GFP protein expression in MKN45, Panc-1 and RWP-1 control (*pLKO)* and *SOX9*-silenced cells (*sh1*) lentivirally transduced with *BMI1* (*pLKO BMI1* and *sh1 BMI1*) or *GFP* (*pLKO GFP* and *sh1 GFP*)(n ≥ 4). β-actin levels were used as a loading control. (**B)** Relative cell growth determined by cell count experiments in RWP-1 *pLKO* and *SOX9*-silenced PDAC cell line (*sh1*) lentivirally transduced with *BMI1* (*pLKO BMI1* and *sh1 BMI1*) or *GFP* (*pLKO GFP* and *sh1 GFP*) (n ≥ 3). (**C)** Apoptosis represented by the percentage of active Caspase-3 positive cells determined by immunofluorescence staining in MKN45, Panc-1 and RWP-1 pLKO and *SOX9*-silenced cells (*sh1*) lentivirally transduced with *BMI1* (*pLKO BMI1* and *sh1 BMI1*) or *GFP* (*pLKO GFP* and *sh1 GFP*) (n ≥ 4). (**D)** Apoptosis represented by the percentage of cleaved PARP1 positive cells determined by immunofluorescence staining in MKN45, Panc-1 and RWP-1 *pLKO* and *SOX9*-silenced cells (*sh1*) lentivirally transduced with *BMI1* (*pLKO BMI1* and *sh1 BMI1*) or *GFP* (*pLKO GFP* and *sh1 GFP*) (n ≥ 4). (**E)** Cellular senescence represented by the percentage of β-Galactosidase (SA β-Gal) positive cells in Panc-1 and RWP-1 *pLKO* and *SOX9*-silenced cells (*sh1*) lentivirally transduced with *BMI1* (*pLKO BMI1* and *sh1 BMI1*) or *GFP* (*pLKO GFP* and *sh1 GFP*) (n ≥ 4). (**F)** Proliferative capacity represented by the percentage of phospho-histone H3 (p-H3) positive cells analyzed by immunosfluorescence staining in MKN45, Panc-1 and RWP-1 *pLKO* and *SOX9*-silenced cells (*sh1*) lentivirally transduced with *BMI1* (*pLKO BMI1* and *sh1 BMI1*) or *GFP* (*pLKO GFP* and *sh1 GFP*) (n ≥ 4). Asterisks (*, ** and ***) indicate statistical significance (p < 0.05, p < 0.01, and p < 0.001, respectively).

The fact that SOX9 modulation impacted on the expression of BMI1 in tumor cells *in vitro* and *in vivo*, suggested that BMI1 could constitute an effector of the pro-tumoral activity of SOX9. To further test this idea, we lentivirally transduced a construct encoding *BMI1* gene in MKN45 GC cells and Panc-1 and RWP-1 pancreatic cancer cells with *SOX9* knockdown or controls. The cells transduced with *BMI1* encoding construct presented BMI1 overexpression in the case of control cells or restoration of BMI1 levels in *SOX9* silenced cells (Fig. 5A). Moreover, at molecular level, BMI1 restoration reduced the expression of p21^CIP^ respect to the levels found in *SOX9*-silenced cells (Fig. 5A).

Then, we evaluated cellular phenotypes in terms of viability, apoptosis, proliferation and senescence *in vitro*. First, we noted that ectopic expression of BMI1 increased the number of tumor cells or restored the number counted in SOX9-silenced cultures (Fig. 5B). Accordingly, ectopic BMI1 also abrogated significantly the induction of apoptosis promoted by *SOX9* silencing, reducing by over 50% the percentage of cells with active Caspase-3 and proteolyzed PARP1 in gastric and pancreatic cancer cells (Fig. 5C,D). Similarly, senescence induction by *SOX9* silencing was significantly mitigated (over 50% reduction) by BMI1 restoration in tumor cells (Fig. 5E). Regarding cell proliferation, our results showed that BMI1 overexpression significantly increased the number of p-H3 positive cells compared to *SOX9* knockdown cells (Fig. 5F). This indicates that BMI1 re-establishes the proliferative capacity of cancer cells abrogated by *SOX9* knockdown.

**Figure 6:**
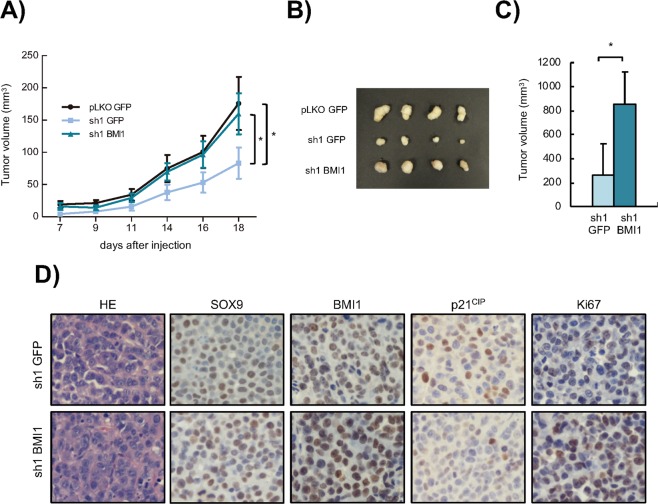
*BMI1* re-expression in cancer cells with *SOX9* knockdown restores tumor growth *in vivo*. **(A)** Volume at the indicated time points of the tumors generated after subcutaneous injection of 1 × 10^5^ MKN45 *pLKO GFP*, *sh1 GFP* or *sh1 BMI1* cells in nude mice (n = 12 injections/ condition). (**B)** Picture of representative tumors generated in (**A**). (**C)** Average volume of the subcutaneous tumors generated by the injection of 0.5 × 10^6^ Panc-1 cells with *SOX9* silencing (*sh1 GFP*) and Panc-1 cells with *SOX9* silencing and *BMI1* restoration (*sh1 BMI1*) in nude mice (n = 8 injections/condition). (**D)** Representative images of the hematoxilin-eosin (HE) staining and immunohistochemical staining of SOX9, BMI1, p21^CIP^ and Ki67 in tumors from (**A**). Asterisks (*, ** and ***) indicate statistical significance (p < 0.05, p < 0.01, and p < 0.001, respectively).

Finally, we tested whether BMI1 is necessary for SOX9 pro-tumoral activity *in vivo*. For this, we inoculated either control *(pLKO)* or SOX9-silenced (*sh1*) MKN45 or Panc-1 cells, which were also transduced with BMI1 or empty vector, to immunodeficient mice. While MKN45 control cell derived tumors grew to almost 200 mm^3^, those derived from *sh1* cells only grew to 50 mm^3^ (Fig. 6A,B). Interestingly, BMI1 restoration completely abrogated the reduction of tumor growth elicited by *SOX9* knockdown (Fig. 6A,B). Similarly, BMI1 restoration also increased the tumor growth capacity of *SOX9* knockdown Panc-1 cells (Fig. 6C). Accordingly, immunohistochemistry of tumors derived from MKN45 cells showed increased number of Ki67 positive cells in *sh1* with *BMI1* restoration compared to *sh1* alone (Fig. 6D). Furthermore, this technique also revealed reduction in the staining of p21^CIP^ in tumors derived form cells with BMI1 restoration (Fig. 6D). Overall, these results show that the ectopic restoration of BMI1 in *SOX9*-silenced cells recovers the aggressive phenotype of tumor cells.

## Discussion

In this study, we show that SOX9 plays a role in cancer progression, not only regulating the activity of CSCs, but also modulating the function of the heterogeneous tumor cells that constitute the tumor bulk. In this context, SOX9 affects a broad plethora of cellular processes that contribute to tumor progression. In particular, SOX9 maintains cell viability, is relevant for cell survival and senescence evasion and promotes proliferation. At the mechanistic level, the transcriptional repressor BMI1 is an important effector of SOX9 in those processes through the negative regulation of *p21*^*CIP*^.

We found that SOX9 activity impacts on cell viability and influences cell proliferation in differentiated pancreatic, glioblastoma and gastric cancer cells. Furthermore, our data reveal that *SOX9* silencing promotes that tumor cells undergo senescence and apoptosis. In agreement with our findings, it has been previously observed that high levels of *Sox9* were sufficient to bypass cellular senescence^33^ and prevented apoptosis in non-tumor cells^36^. Our results highlight the function of SOX9 in controlling multiple processes associated to cancer and, hence explaining, how SOX9 potentiates tumor progression not only regulating the activity of CSCs.

BMI1 is an important epigenetic regulator, which restricts cell proliferation and mediates senescence and apoptosis during homeostasis^37^. In cancer, its levels are commonly elevated and it plays a potent oncogenic role in multiple types of cancer including pancreatic, glioblastoma and gastric cancer^38–40^. In this work, we found that modulation of SOX9 levels affects BMI1 expression in multiple cancer cell lines *in vitro* and *in vivo*. These results are in agreement with previous studies that linked SOX9 and BMI1 in primary fibroblasts and colorectal CSCs^23,33^. This regulation is likely to be direct since chromatin immunoprecipitation experiments have shown that SOX9 binds to the promoter of BMI1 in different types of cells and contexts^33,41^. Moreover, our results show that there is also a positive correlation between *SOX9* and *BMI1* expression, and negative between *SOX9* and *p21*^*CIP*^ in clinical samples. In line with this, previous studies analyzed the correlation between SOX9, BMI1 and p21^CIP^ in different types of cancers and showed independently that SOX9 regulates proliferation through a positive correlation with BMI1 and an inverse correlation with p21^CIP^ expression^23,31,33–35^. Together, these results highlight that BMI1 is a relevant mediator of SOX9 in promoting tumor malignancy and show the importance of this axis in cancer progression, thus providing new therapeutic possibilities, such as BMI1 inhibition, a strategy that is being currently evaluated in different cancer clinical trials. Thus, the trial NCT03761095 is aimed at evaluating the safety of the orally active BMI1 inhibitor PTC596 (PTC Therapeutics) in combination with dacarbazine for the treatment of advanced leiomyosarcoma. Moreover, also the trials NCT03206645 and NCT03605550 are currently evaluating this inhibitor. The first is a dose-escalation study oriented to evaluate its safety, tolerability and pharmacokinetics in combination with conventional chemotherapy for the treatment of ovarian and fallopian tube cancer or primary peritoneal cancer. The latter tries to determine the safe dose of PTC596 for its administration in combination with radiation in children with newly diagnosed diffuse intrinsic pontine glioma and high-grade glioma.

**Figure 7:**
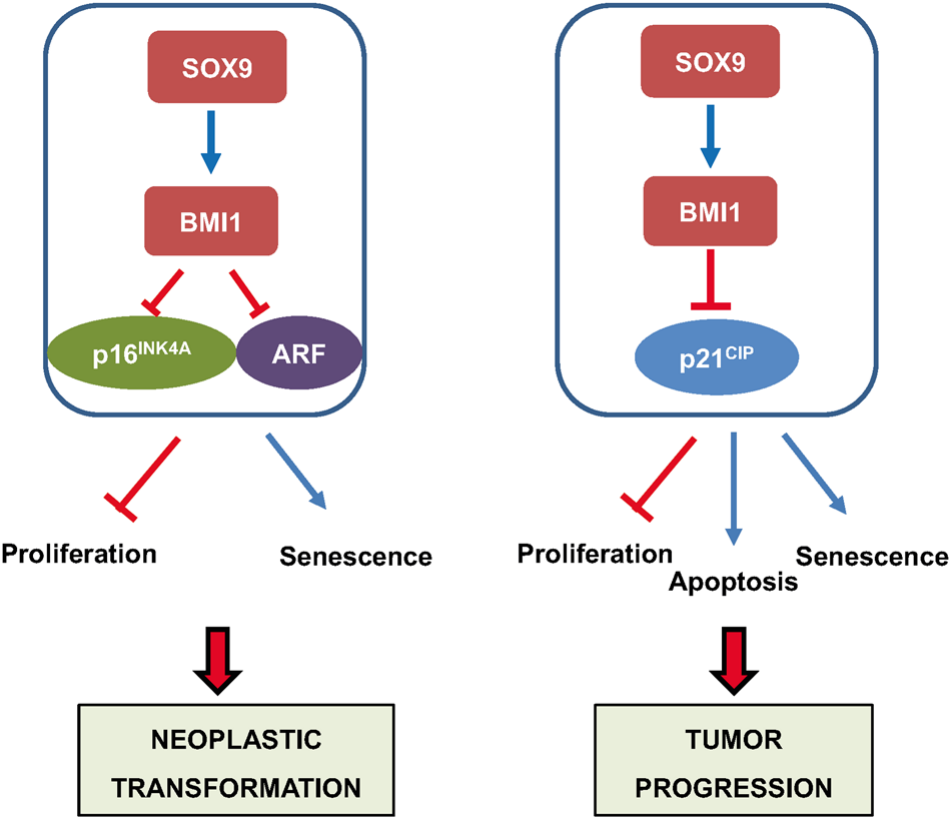
Pro-tumoral action and molecular mechanism of SOX9 in cancer. In the context of non-tumor cells (left panel), SOX9 is able to promote neoplastic transformation through the induction of cell proliferation and the evasion of senescence. At the molecular level, this transformation takes place through the induction of BMI1 expression, which represses the expression of the tumor suppressors *p16*^*INK4A*^ and *ARF* (previously described by Matheu *et al*., 2012). In the context of tumor cells (right panel), generally deficient in *p16*^*INK4A*^ and *ARF* expression, SOX9 enhances tumor progression through the promotion of cell proliferation and the evasion of apoptosis and senescence. In this context, the molecular mechanism involves the repression of the tumor suppressor *p21*^*CIP*^.

BMI1 represses the transcription of multiple genes including relevant tumor suppressor genes such as *p21*^*CIP *^^42–44^, as well as the *Ink4a/Arf* locus, encoding *p14*^*Arf*^ and *p16*^*INK4a*^, being the latter the best-known target in cell growth arrest and senescence^37^. Indeed, we have previously shown that *Sox9* promotes proliferation and favors neoplastic transformation of primary cells through *Bmi1*, whose upregulation consequently represses the expression of *Ink4a* and *Arf* genes^33^. *Ink4a/Arf* locus is frequently mutated and inactivated in human cancers^45^, as it is the case also of many tumor cell lines including the ones used in this study. Thus, the action of SOX9-BMI1 axis in driving processes associated to tumor progression is not attributable to the repression of *p16*^*INK4*a^ and/or *p14*^*Arf*^. Indeed, our results show that this axis promotes tumor cell survival and proliferation via *p21*^*CIP*^, since the restoration of BMI1 levels in *SOX9* silenced cells, also modulated, in this case inhibited, the expression of this tumor suppressor. Thus, we postulate that the axis SOX9-BMI1 plays a relevant role in the different stages of cancer initiation and progression, in which they inhibit the expression of tumor suppressors sequentially. The repression of *Ink4a/Arf* locus is required at early stages of neoplastic transformation and tumor formation, whereas the repression of *p21*^*CIP*^ is necessary in the action exerted by the axis SOX9-BMI1 for cancer progression (Fig. 7).

In summary, our findings show that SOX9 is highly relevant in the survival of population of cells constituting the tumor bulk in multiple types of cancer contributing to evasion of apoptosis. Moreover, we reveal that SOX9 controls the proliferative capacity of tumor cells and facilitates evading senescence. Mechanistically, SOX9 exerts these pro-tumoral actions through BMI1- p21^CIP^, providing novel knowledge regarding the molecular events leading to cancer progression.

## Experimental Procedures

### Human subjects

Clinical information of glioblastoma patients was obtained from the Donostia University Hospital. Human glioma samples were provided by the Basque Biobank for Research-OEHUN (http://www.biobancovasco.org). All study participants signed informed consent form approved by the Institutional Ethical Committee. The study was approved by the ethic committee of Euskadi (PI2016151). All methods were performed in accordance with the relevant guidelines and regulations.

### Cell culture

Pancreatic carcinoma cell lines (Panc-1, RWP-1, IMIMPC-2 and BxPC-3) were kindly provided by Dr. Real (CNIO) and Dr. Navarro (IMIM-Medical Research Institute). Glioblastoma cell lines U251MG (U251), U87MG (U87) and U373MG (U373) were purchased from the ATCC (American Type Culture Collection). The human gastric adenocarcinoma AGS and MKN45 cell lines were gifts from Dr. Haas (Ludwig-Maximilians-Universität München, München, Germany). All cell lines were mycoplasma free. Cells were cultured as adherent monolayers at 37 °C and 5% CO_2_ in DMEM medium (Gibco) (MKN45 cells in RPMI) supplemented with 10% FBS (Gibco), L-glutamine (2 mM), 100 U/ml penicillin and 100 µg/ml streptomycin.

### Gene silencing and overexpression

For *SOX9* silencing by shRNA, cells were infected with lentivirus harboring the sh*SOX9* plasmid #40644 and the corresponding pLKO.1 puro control plasmid #8453 from Addgene, gifts from Dr. Bob Weinberg. Transduced cells were selected 48 hours later in the presence of 2 μg/mL puromycin for 48–72 hours. For lentiviral *SOX9* overexpression the pWXL-SOX9 plasmid #36979 from Addgene, gift from Bob Weinberg, was used. The pWXL plasmid #12257 from Addgene, gift from Didier Trono, was used as control. For *BMI1* upregulation cells were infected with lentivirus harboring the plasmid pLenti CMV GFP Puro-Bmi1 (gift from Jacqueline Lees). Lentiviral infections were performed as previously described^31^. All infections were performed at a MOI of 10 for 6 hours.

### Western blot

Immunoblots were performed following standard procedures. Primary antibodies used were: SOX9 (AB5535, Millipore), BMI1 (05-637, Millipore), p21^CIP^ (sc-397-G, Santa Cruz Biotechnology), GFP (ab6673, abcam) and β-actin (AC-15, Sigma). Primary antibodies were detected with HRP-linked secondary antibodies: anti-rabbit (7074S, Cell Signaling Technology), anti-mouse (7076S, Cell Signaling Technology) and anti-goat (sc-2020, Santa Cruz Biotechnology). Protein bands were detected using the ECL system (NOVEX® ECL, Invitrogen).

### Immunofluorescence

For immunofluorescent detections, cells were seeded in Lab-Tek II Chamber Slides (ThermoFisher Scientific) and fixed with 4% paraformaldehyde for 15 min at RT. Then, cells were incubated with PBS supplemented with 0.3% Triton X-100 and 5% FBS for 1 hour at RT. Cells were incubated overnight at 4 °C with different primary antibodies: phospho-histone H3 (phospho S10) (ab14955, Abcam), Cleaved PARP1 (ab32064, Abcam) and Active Caspase 3 (AF835, R&D Systems). Secondary antibodies conjugated to fluorochromes were incubated for 1 hour at RT and chromatin staining was performed with Hoechst 33342 (Molecular Probes). Slides were mounted with Fluoro-Gel mounting medium (Electron Microscopy Sciences) and preparations were visualized and documented using a Nikon Eclipse 80i microscope.

### β-Galactosidase activity

To analyze cellular senescence, β-Galactosidase activity was measured in cells using the Senescence β-Galactosidase Staining Kit of Cell Signaling Technology (#9860), according to the manufacturer’s protocol. Briefly, cells were fixed and incubated overnight at 37 °C in a dry incubator with a staining solution containing X-Gal. Cells were observed in an inverted light microscope and different views were captured randomly to calculate the positive staining rate for each experimental condition.

### mRNA expression analysis

Total RNA was extracted with trizol (Life Technologies). Reverse transcription was performed using the High-Capacity cDNA Reverse Transcription Kit (ThermoFisher) according to the manufacturer’s guidelines. Quantitative real-time PCR was performed in an ABI PRISM 7300 thermocycler (Applied Biosystems) using Power SYBR® Green Master Mix (ThermoFisher), 10 mmol/L of primers and 20 ng of cDNA. GAPDH was used as housekeeping gene. The ΔΔCT method was used for relative quantification.

### Computational biology analysis

TCGA data were obtained from https://www.cancer.gov/tcga and https://r2.amc.nl. All the software and graphs for transcriptomic analysis were developed using in-house code developed in MATLAB.

### *In vivo* carcinogenesis assays

All animal handling and protocols were approved by the animal care ethic committee of Biodonostia Institute and were conducted in conformity with the EU guidelines and regulations for animal experimentation. For subcutaneous injection, cells were harvested with trypsin/EDTA and resuspended in PBS. 1 × 10^5^ MKN45 cells and 0.5 × 10^6^ Panc-1 cells were injected into both flanks of 8 week-old Foxn1^nu^/Foxn1^nu^ mice. External calipers were used to measure tumor size, from which tumor volume was estimated by V = L*W^2^*0.5, where L is the tumor length and W is the tumor width.

### Hematoxylin and eosin staining and immunohistochemistry

Tumors generated in mice were dissected, fixed in 10% formalin for 48 hours and embedded in paraffin. 4 µm thick sections were stained with hematoxylin and eosin (H&E). For immunohistochemistry, sections were rehydrated and heated in citrate buffer pH 6 for antigen retrieval. Antibodies used for detections included SOX9 (AB5535, Millipore), BMI1 (05-637, Millipore), p21^CIP^ (sc-6246, Santa Cruz Biotechnology) and Ki67 (ab15580, Abcam). The stainings were developed with 3,3′ Diaminobenzidine (DAB) and nuclei were counterstained with hematoxylin.

### Data evaluation

Data are presented as mean values ± standard error (SEM), with the number of experiments (n) in parentheses. Unless otherwise indicated, statistical significance (p-values) was calculated using the Student´s t -test. Asterisks (*, ** and ***) indicate statistical significance (p < 0.05, p < 0.01, and p < 0.001, respectively).

## Supplementary information

Supplementary information.
