# Supplementary material for: SOX9 promotes tumor progression through the axis BMI1-p21CIP
Source: Sci Rep. 2020 Jan 15;10:357. doi: 10.1038/s41598-019-57047-w (PMC6962164; doi:10.1038/s41598-019-57047-w)

Supplementary information file

SOX9 promotes tumor progression through the axis BMI1-p21^{CIP}

Paula Aldaz[1,#], Maddalen Otaegi-Ugartemendia[1,#], Ander Saenz-Antoñanzas[1], Mikel Garcia-Puga[1], Manuel Moreno-Valladares[1,2], Juana M. Flores[3], Daniela Gerovska[4], Marcos J Arauzo-Bravo[4,5,6], Nicolas Samprón[1,2,5], Ander Matheu[1,5,6,*], Estefania Carrasco-Garcia[1, *]

[1] Cellular Oncology Group, Biodonostia Health Research Institute, San Sebastian, Spain.

[2] Donostia Hospital, San Sebastian, Spain.

[3] Department of Animal Medicine and Surgery, Complutense University of Madrid, Spain.

[4] Computational Biology and Systems Biomedicine Group, Biodonostia Health Research Institute, San Sebastian, Spain

[5] CIBERfes, Spain.

[6] IKERBASQUE, Basque Foundation, Bilbao, Spain.

[#] *These authors have contributed equally and should be considered as co-first authors*

**Corresponding authors*:

Ander Matheu, PhD

Biodonostia Institute, Paseo Dr. Beguiristain s/n, San Sebastian, Spain

Tel.: +34.943 006073, Fax: +34. 943 006250, Email: ander.matheu@biodonostia.org

Estefanía Carrasco- García, PhD

Biodonostia Institute, Paseo Dr. Beguiristain s/n, San Sebastian, Spain

Tel.: +34.943 006296, Fax: +34. 943 006250, Email: estefania.carrasco@biodonostia.org

Supplementary information file

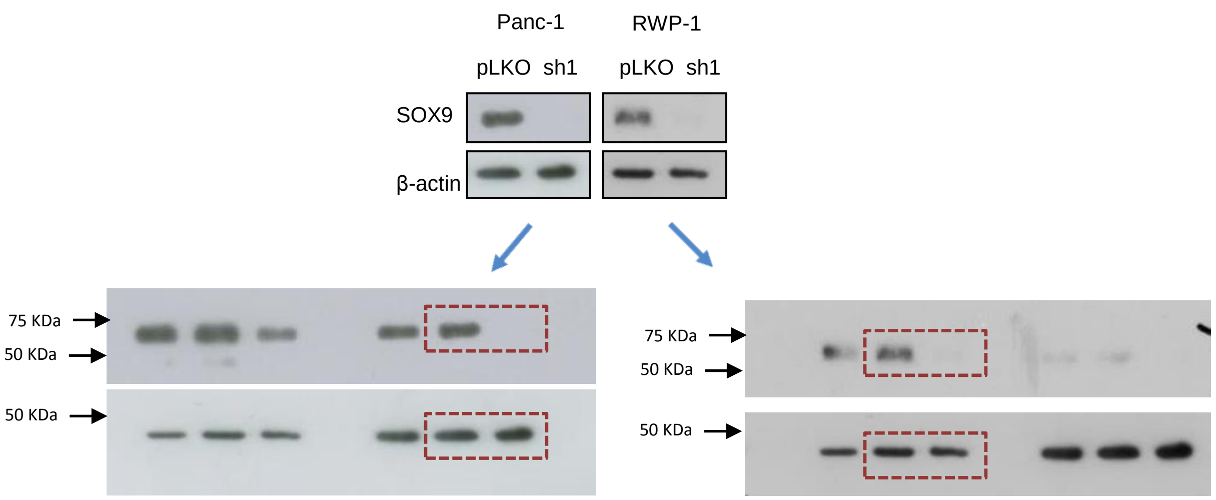

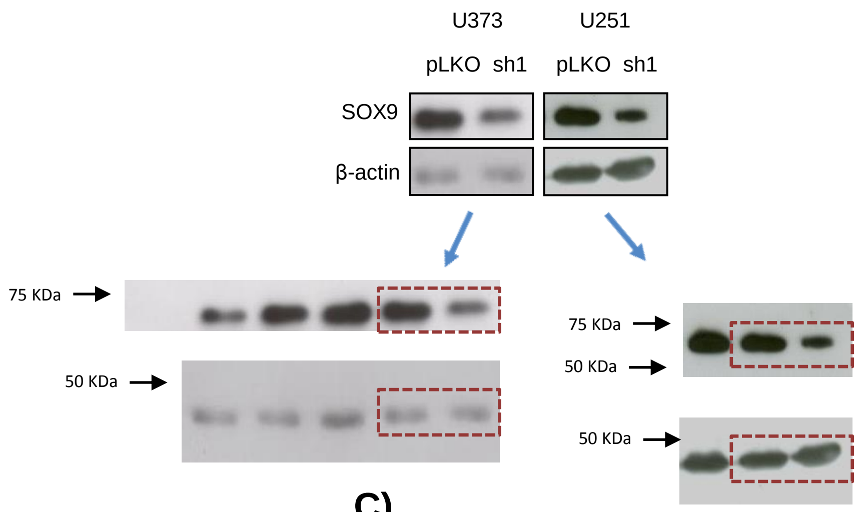

C)

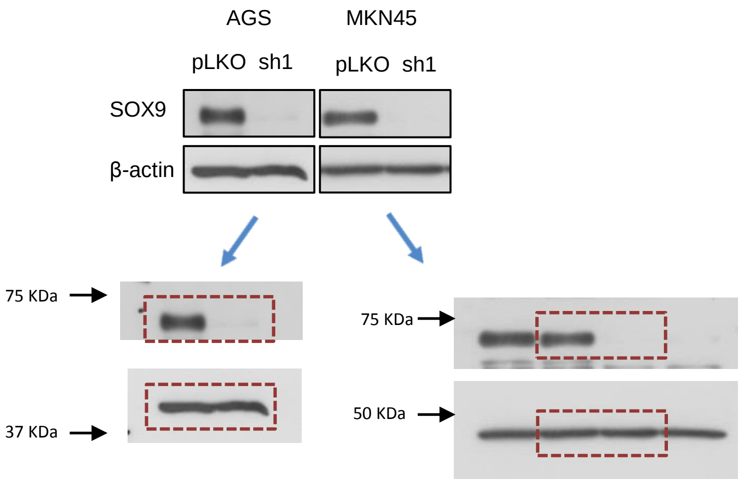

Supplementary information file

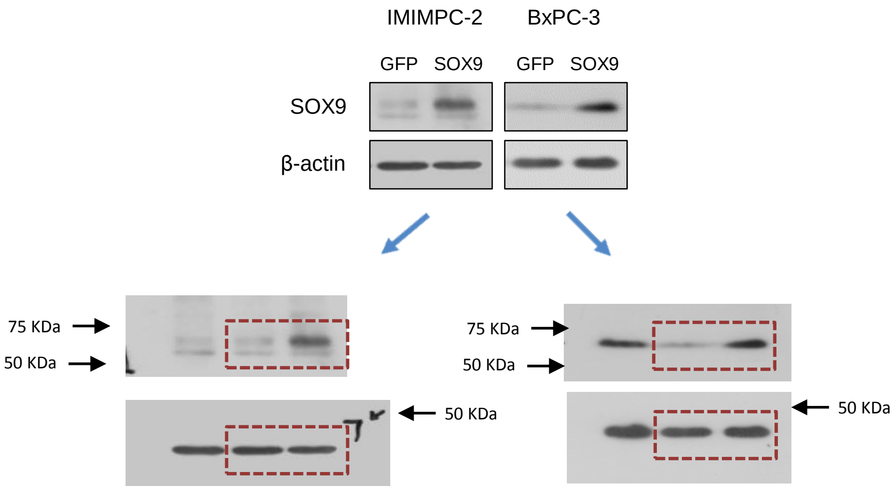

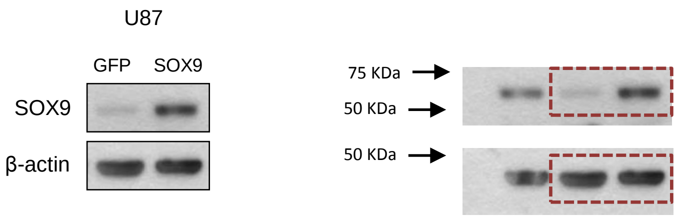

Supplementary information file

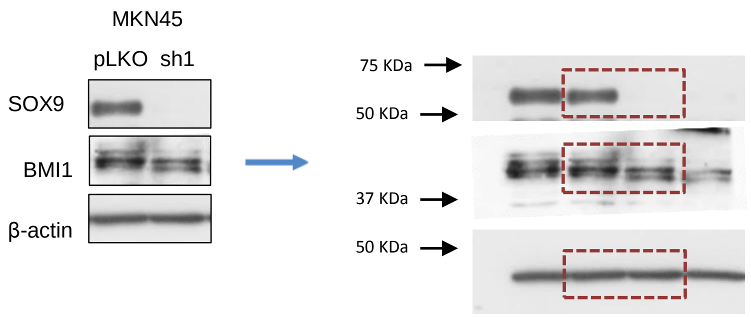

Supplementary information file

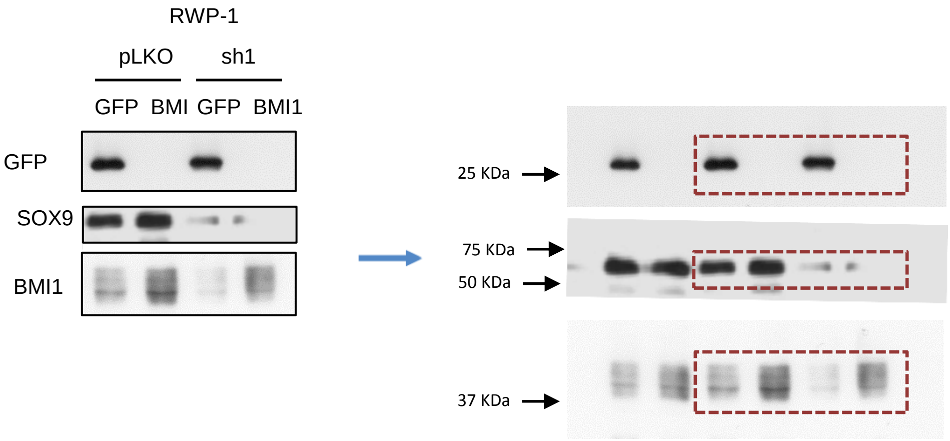

Supplement: Supplementary file 1 — Supplementary information. [file 41598_2019_57047_MOESM1_ESM.pdf]
